# Quality-of-life and detailed functional outcome after IONM-aided microsurgical resection of cervical and thoracic intramedullary spinal cord tumors in adults

**DOI:** 10.1007/s00701-026-06836-0

**Published:** 2026-03-25

**Authors:** Sebastian Siller, Sylvain Duell, Deniz Reyhaniye, Julian Kramer, Patrick N. Harter, Florian Ringel, Stefan Zausinger, Joerg-Christian Tonn, Andrea Szelenyi

**Affiliations:** 1 Department of Neurosurgery, University Hospital Regensburg, Franz-Josef-Strauss-Allee 11, 93053 Regensburg, Germany; 2 Department of Neurosurgery, LMU Hospital, Ludwig-Maximilians-University Munich, Campus Großhadern, Marchioninistrasse 15, 81377 Munich, Germany; 3 Center for Neuropathology and Prion Research, LMU Hospital, Ludwig-Maximilians-University Munich, Campus Großhadern, Marchioninistrasse 15, 81377 Munich, Germany; 4 German Cancer Consortium (DKTK), Partner Site Munich, Pettenkoferstrasse 8a, 80336 Munich, Germany; 5 German Cancer Research Centre (DKFZ), Im Neuenheimer Feld 280, 69120 Heidelberg, Germany; 6 Bavarian Cancer Research Center (BZKF), Östliche Stadtmauerstrasse 30, 91054 Erlangen, Germany; 7 Wirbelsaeulentherapie am Rindermarkt, Rindermarkt 16, 80331 Munich, Germany

**Keywords:** Intramedullary spinal cord tumor surgery, IMSCT, Quality-of-life, Outcome, Neurological status, Functional status

## Abstract

**Background/Purpose:**

Resection is the therapy of choice for cervicothoracic intramedullary spinal cord tumors (ctIMSCTs). While coarse neurological outcome has been multiply reported, more detailed data on longer-term outcome of quality-of-life (QoL) aspects as well as functional and neurological status are still scarce.

**Method:**

We prospectively assessed all patients undergoing ctIMSCT surgery with IONM in our neurosurgical center between 2017 and 2023. Detailed neurological and functional status as well as McCormick Score, Barthel Index (BI) and physical & mental QoL via Short-Form-36-Health-Survey Score (SF36-P/MCS) were assessed pre- and immediately postoperatively and regularly during long-term follow-up (median: 25 months) and correlated with patients’ and tumor characteristics.

**Results:**

40 patients (m/f: 26/14) with a median age of 47 years were enrolled. In 78% and 22% of the patients tumors were located in the cervical and thoracic spine. Preoperatively, motor deficits were present in 40%, sensory disturbances in 78% and gait ataxia (proprioceptive deficits) in 38%; median McCormick Score was 1, mean SF36-PCS and SF36-MCS were 45.1 and 43.3, and median BI was 100 in the overall cohort. Gross-total resection rate was 88%. At last follow-up, 80% of the patients had a postoperative improved/stable course in motor, 25% in sensory and 75% in gait function compared to the preoperative status. The overall cohort’s median McCormick-Score was 1, while the mean SF36-PCS and SF36-MCS were 44.5 and 44.5 and the median BI was 100 (each with no significant differences compared to the preoperative status). Rates for individual improvement/stability/deterioration were 30%/33%/37% for physical QoL aspects and 33%/35%/32% for mental QoL aspects. Presence of a permanent postoperatively new/worsened sensory deficit (OR = 0.08; *p* = 0.01) and a preoperatively higher SF36-PCS (OR = 0.90; *p* = 0.01) were significantly associated with a worse follow-up outcome regarding physical QoL perception.

**Conclusions:**

Quality-of-life and motor/gait function improves/stabilizes in the majority of patients during follow-up after ctIMSCTs resection. Persistence of a postoperative deterioration in sensory function and a higher preoperative QoL perception are factors associated with a worse physical QoL follow-up outcome.

**Supplementary Information:**

The online version contains supplementary material available at 10.1007/s00701-026-06836-0.

## Introduction

Intramedullary spinal cord tumors (IMSCTs) are rare and comprise 2–4% of central nervous system neoplasms and 20–30% of all spinal cord tumors in adults [1, 18]. Resection is the mainstay of treatment today and plays a critical role to prevent further permanent neurological deficits, while radical resection has additionally been associated with increased survival in certain entities [5, 6, 8, 16, 27]. For most IMSCT entities long-term progression-free survival is possible which underlines the importance of the concept of maximally safe microsurgical tumor resection. While recent advances in neuroimaging, microsurgical techniques, and intraoperative neurophysiological monitoring have greatly facilitated safer and more aggressive resections [15, 17], surgery still poses significant technical challenges and the risk of perioperative and long-term morbidity [6, 8, 16].

So far, several studies have described clinical and functional outcomes after IMSCT resection in the short- and longer-term follow-up, mostly utilizing gross-functional compound scores (e.g. McCormick scale) in a retrospective setting [3, 6–8, 16, 22, 27]. However, none of these studies has specifically addressed the detailed neurological as well as fine-motor/-functional outcome exceeding the graduation into coarse compound scores (e.g. McCormick scale) or the mere dichotomization in ambulatory or not. Additionally, in terms of quantification of functional outcome, the literature is still highly scarce for health-related quality of life (QoL) aspects before and after IMSCT resection and the available evidence from limited retrospective studies remains highly controversial [13, 19, 20, 23, 26]. In particular, no data from prospective studies are yet available integrating a multidimensional approach in patients’ evaluation with detailed neurological, functional and QoL evaluation before and after surgery and along the postoperative follow-up period. These data, however, would be essential for a better prediction of health-related outcome after IMSCT surgery and for an optimized decision finding for patients and treating physicians.

Our study is the first to elucidate detailed neurological, functional and QoL aspects both prior to IMSCT surgery as well as in the postoperative short- and longer-term course in a prospective manner. Hence, we performed this prospective study to clarify whether patients after IMSCT resection improve or stabilize during the postoperative long-term follow-up in terms of.

- neurological status,
- daily-life function, and
- physical and mental QoL perception (and which factors might be of influence).

## Methods and materials

All patients had been referred to our institute (Department of Neurosurgery, Ludwig-Maximilians-University Hospital, Munich, Germany) from April 2017 to July 2023 for multimodal IONM-aided microsurgical resection of an IMSCT in the cervical or thoracic spine (excluding conus medullaris tumors). After study approval by the local Institutional Review Board (AZ17-145), these patients were prospectively enrolled following informed consent. We prospectively collected and analyzed epidemiological aspects, clinical characteristics, imaging findings, management strategies, operative records, complications, and detailed individual short- and longer-term outcomes for each single patient as described below.

### Detailed neurological, quality-of-life and functional evaluation

Detailed neurological assessment (including sensory, reflex, muscle tone and gait examinations) as well as fine-motor and functional evaluation via the Medical Research Council (MRC) grading system for muscle strength [2] was performed in all patients before and after surgery as well as at the 3-, 12- and 24-months as well as at last follow-up timepoints (median: 25 months after surgery, range: 15–67 months). Postoperatively new or worsened sensorimotor deficits, pain or painful dysaesthesia and gait ataxia as well as bladder/bowel dysfunction were additionally recorded. Impairment of epicritical sensibility aspects (especially superficial aesthesia) was subsumed as ‘sensory deficits’, while impairment or loss of proprioception was mainly assessed by a challenged gait examination (including walking with tandem gait and on toes/heels with and without eyes closed among others) and subsumed as ‘gait ataxia’.

The classification of McCormick [12] was used for gross-functional evaluation, while the Short Form (SF)−36v2® Health Survey [25] and the Barthel Index (BI) [11] were used for the assessment of quality-of-life aspects and general performance in daily life before and after surgery as well as at every follow-up visit. The Odom score [14] and Patient Satisfaction Index (PSI) [4] were used to evaluate the general postoperative and follow-up outcome and subjective satisfaction.

With regard to the SF-36v2® Health Survey, the Physical Compound Score (PCS) was used to described the physical aspects of QoL perception, while the Mental Compound Score (MCS) was used to describe the mental aspects of QoL perception. For SF36-PCS and SF36-MCS the minimal important clinical difference to determine ‘improvement’ or ‘deterioration’ was a score change of > 5 points. If changes in SF36-PCS and SF36-MCS were < 5 points, we described the score as ‘stable’.

As a novel category we introduced the timepoint of ‘best postoperative status’ as an addition to the above defined fixed-time endpoints. For the category ‘best postoperative status’, we used that individual patient’s follow-up timepoint after surgery when the postoperative neurological, functional and QoL outcomes of that individual patient were most favorable; in median, the timepoint of ‘best postoperative status’ was achieved at 12 months (range 3–50 months) after surgery but this was highly different between the individual patients.

### Imaging evaluation

Evaluation of tumor location, spinal level and distribution within the spinal cord were based on preoperative contrast-enhanced MR imaging. On the basis of radiographical and intraoperative observations, tumors were characterized as either dorsal or ventral of the coronary spinal cord midline and as either left- or right-dominated of the sagittal spinal cord midline or central; all tumors were located completely intramedullary (including both cases with the rare occurrence of completely intramedullary schwannomas). Gross-total resection was defined as complete tumor removal according to intraoperative microscopic findings and postoperative contrast-enhanced T1- and T2-weighted MR imaging. The presence and extent of edema and syringomyelia were assessed using T2-weighted and/or FLAIR MR imaging sequences.

### Surgical procedures & neuropathological assessment

Anesthesia was performed with total intravenous anesthesia, carefully avoiding the application of muscle relaxants despite for intubation purposes. All patients were administered steroids preoperatively. For tumor resection, patients were placed in prone position. Via a posterior midline approach, the lamina and spinous processes overlying the tumor were exposed. To provide exposure of the tumor margins, a minimal-invasive approach in terms of a unilateral laminotomy or hemilaminectomy was performed whenever possible. If exposure of tumor margins required a bilateral approach, laminectomy was performed for single-level lesions and laminoplasty were for two-level or more level lesions. In all cases intraoperative ultrasonography was used before dural opening to assure a precise exposure of the tumor.

If IMSCTs did not approach the spinal cord surface to serve as entry point for intramedullary tumor resection, a midline myelotomy was performed by sharp dissection after visual identification and marking of the anatomical midline by the surgeon and confirmatory recording of both spinal somatosensory evoked potentials (SSEPs) to follow tibial and/or median stimulation with an 8-channel dorsal column mapping (DCM) -electrode (AdTech Co., USA) as well as spinal cord stimulation by bipolar concentric probe (Inomed Co., Germany) with recording of cortical SSEP phase reversal at C3/C4 as previously described [24]. Resection was performed under microscope- and ultrasound-guidance with microsurgical techniques under continuous multimodal IONM of both SSEPs and muscular transcranially motor evoked potentials (mTcMEPs) as well as Direct (D) wave and free-running electromyography (EMG) utilizing an integrated IONM system (ISIS, Inomed Co., Germany) as previously described [21]. Any IONM changes were immediately issued to the surgeons’ team and the interpretation was performed interdisciplinary between the surgical and electrophysiological team. Significant reduction in SSEPs amplitude ≥ 50% and/or an increase in SSEPs latency ≥ 10% as well as loss or significant decrement in mTcMEPs amplitude ≥ 80% or in D Wave amplitude ≥ 50% as well as significant spontaneous EMG activity (e.g. neurotonic discharges) especially during or immediately after surgical manipulation were defined as ‘warning criteria of IONM’ and ‒ if technical reasons (e.g. dislocation of electrodes), anesthesiological reasons (e.g. lowering of blood pressure or body temperature, change of intravenous anesthesia management or addition of volatile anesthetics) and temporary surgical reasons (e.g. irrigation with cold saline solution) are excluded ‒ classified as ‘significant IONM changes’. In circumstances of ‘significant IONM changes’, immediate corrective actions were initiated, e.g. cessation of additional volatile anesthetics, correction of blood pressure or modification of the surgical technique (e.g. temporary haltering of resection, reduction of traction on the tumor or surrounding tissue, irrigation with warm saline solution and/or continuation of resection at distant sites). In case of repetitive (or persistent) ‘significant IONM changes’ during the resumption of tumor resection/dissection in the very same area where prior manipulation had been halted for corrective actions due to critical IONM changes, further attempts for additional tumor resection/dissection were abandoned. After termination of resection, the dura was closed in a watertight manner and a subdural hematoma was excluded by a subsequent intraoperative ultrasonographic control before tissue and wound closure.

Neuropathological (re)assessment was performed according to the 2021 CNS 5 WHO classification for central nervous system tumors [10]. The final diagnoses reported here are based on the combination of histology, genetics and (where available) methylome classifier results. Adjuvant treatment concepts (radiation, chemotherapy, chemoradiotherapy, etc.) were applied whenever indicated by the local interdisciplinary tumor board.

### Statistical analysis

Statistical analysis was performed using Sigma Plot for Windows v.11 (Systat Software Inc., USA). Differences are defined to be statistically significant if the p value is < 0.05. For comparison of groups for differences the Student’s t-test was used for numeric values, the Mann–Whitney Rank Sum test for ordinal variables and the χ2-test respectively Fisher’s exact test (in case of 2 × 2-contingency tables) for nominal variables. Assumptions for parametric tests were tested beforehand. To control for confounding variables and to identify independent risk factors associated with QoL (SF36) outcomes, logistic regression analyses (polytomous variables) and χ2-test respectively Fisher’s exact tests (dichotomous variables) were performed. To avoid overfitting, we focused on univariate regression analysis to explore forvariables associated with QoL changes. Only variables demonstrating association (*p* < 0.20 via univariate regression) with QoL changes were additionally analyzed via multivariate regression analyses and displayed in Supplementary Table 6. In Supplemetary Table 1, only permanent ‘significant IONM changes’ in SSEPs, mTcMEPs, D wave and/or frEMG monitoring during surgery with persistence after dura closure were collected and associated with postoperatively new or worsened neurological deficits at last follow-up matching the respective IONM modality.

## Results

### Patients’, tumor and operative characteristics

Altogether 40 patients with IMSCTs were consecutively enrolled and surgically treated in our center during the 6-years observational period. Median age was 47 years and there was a predominance of the male gender (male/female: 1.9:1). Detailed baseline patients’ and tumor characteristics are displayed in Table 1.

**Table 1:** Baseline characteristics at admission for surgery*

Characteristics	Patients
Gender, male/female	26/14
Median age, yrs (range)	47 (18–75)
Comorbidities**, no. (%)	
Cardiovascular diseases	3 (7.5)
Peripheral neuropathy	3 (7.5) [carpal tunnel syndrome]
History of smoking	2 (5.0)
Obesity	4 (10.0)
BMI, kg/m^2^	26.9 ± 4.4
Preoperative symptoms, no. (%)	
Motor deficit	16 (40.0)
Spasticity	6 (15.0)
Sensory deficit	31 (77.5)
Gait ataxia	15 (37.5)
Bladder/bowl dysfunction	8 (20.0)
Pain	20 (50.0)
Mean duration of symptoms, mos	33.3 ± 44.9
Tumor entity***, no. (%)	
Spinal ependymoma, WHO °II	20 (50.0)
Pilocytic astrocytoma, WHO °I	2 (5.0)
Diffuse midline glioma, H3K27-altered, WHO °IV	1 (2.5)
Diffuse leptomeningeal glioneuronal tumor	1 (2.5)
Low-grade neuroepithelial tumor with FGFR3/TACC3 fusion	1 (2.5)
Schwannoma, WHO °I	2 (5.0)
Hemangioblastoma, WHO °I	5 (12.5)
Cavernoma	3 (7.5)
Other	5 (12.5) [capillary hemangioma, neuroma, 3 × not classifiable]
Location of tumor, no. (%)	
High-cervical (C1 – C4 level)	21 (52.5)
Low-cervical (C5 – C8 level)	10 (25.0)
High-thoracic (Th1 – Th6 level)	5 (12.5)
Low-thoracic (Th7 – Th12 level)	4 (10.0)
Extent of solid tumor, no. (%)	
Singel-level	31 (77.5)
Two-level	6 (15.0)
Three-level or more	3 (7.5)
Peritumoral edema, no. (%)	20 (50.0)
Syrinx, no. (%)	16 (40.0)

* Mean values are presented ± standard deviation

**None of the patients suffered from diabetes mellitus, polyneuropathy or alcohol abuse

***based on the 2021 WHO classification of CNS tumors^{#37}^

For exposure of tumor margins a laminotomy was performed in 13% of the cases, hemilaminectomy in 13%, laminectomy in 32% and laminoplasty in 42%. Gross-total resection was accomplished in 88% of the cases according to intraoperative findings and postoperative contrast-enhanced MRI. Mean operative time was 373 ± 81 min. and mean blood loss 450 ± 430 ml. Mean in-patient stay was 8 ± 5 days.

There were no postoperative surgical complications in the overall cohort.

### Detailed neurological, functional and quality-of-life outcome

Detailed neurological outcomes at discharge as well as at 3-, 12-, 24-months and last follow-up timepoint (median: 25 months after surgery, range: 15–67 months) are shown in Table 2. Postoperatively new or worsened motor deficits occurred in 35% of the patients, which were permanent in 57% of those cases. All permanent postoperatively new or worsened motor deficits were mild (deterioration in motor function of MRC grade 1 or 2) and there was no case with a postoperative new plegia. Within those patients with a permanent postoperatively new or worsened motor deficit, 71% of the patients had a stable deficit while 29% showed a further worsening (e.g. due to progression of the underlying spinal cord tumor disease or gliosis formation) during follow-up. In contrast, in 43% of cases with new or worsened motor deficits directly postoperative these symptoms resolved during follow-up. Therefore, the overall rate of the patients with an improved or stable motor function at the last follow-up timepoint was 80%.

**Table 2:** Detailed postoperative neurological outcome during follow-up

Change in (compared to the preoperative status)	Timepoint					
	postop (*n* = 40)	3-mo FU (*n* = 40)	12-mo FU (*n* = 40)	24-mo FU (*n *= 36)	last FU (*n *= 40)	‚best ‘ postop status (*n* = 40)
Motor function						
Improved, %	5%	18%	29%	12%	15%	29%
Stable, %	60%	56%	57%	62%	65%	57%
Deteriorated, %	35%	26%	14%	26%	20%	14%
Sensory function						
Improved, %	8%	21%	18%	19%	20%	33%
Stable, %	22%	5%	21%	8%	5%	21%
Deteriorated, %	70%	74%	61%	73%	75%	46%
Gait ataxia (proprioception)						
Improved, %	10%	21%	21%	23%	20%	30%
Stable, %	48%	58%	54%	46%	55%	58%
Deteriorated, %	42%	21%	25%	31%	25%	12%
Bladder/bowl function/continence						
Improved, %	13%	18%	14%	19%	15%	20%
Stable, %	74%	82%	86%	81%	85%	80%
Deteriorated, %	3%	0%	0%	0%	0%	0%

Sensory changes were the most frequent permanent postoperatively new neurological deficits with a rate of 70% and remained permanent in 87% thereof. They mainly comprised of superficial hypaesthesia and impairment of fine epicritical sensibility, while new or worsened spinal gait ataxia (proprioceptive impairment) was seen in 42% of the patients (which were permanent in 60% of the cases). During follow-up, permanent postoperatively new or worsened sensory deficits were stable in 70% but secondarily worsening in 30% (e.g. due to progression of the underlying spinal cord tumor disease or gliosis formation) of the affected patients, while permanent postoperatively new or worsened gait deficits were stable in all patients without secondary deficit progression. There was no patient with a postoperative deterioration of bladder/bowl function/continence. At last follow-up, 20% of the patients showed an improvement (compared to the preoperative status) of sensory function and 25% of gait stability and 15% of bladder/bowl functions, while gait and bladder/bowl functions were stable in 55% and 85%. The presence of a preoperative tumor-associated syrinx was not statistically associated with the postoperative and follow-up outcome of the sensory function.

Frequency of significant and persisting IONM changes during surgery and their association with modality-matching new or worsened neurological deficits at last follow-up are displayed in Supplementary Table 1.

At the timepoint of the individual patient’s best postoperative status, the rate of patients with improvement/stability (compared to the preoperative status) was 29%/57% for motor function, 21%/33% for sensory function, 30%/58% for gait function and 20%/80% for bladder/bowl function.

Postoperative clinical outcome (Odom Score) and subjective patients’ satisfaction (PSI) during follow-up are shown in Table 3. At last follow-up, about one third of the patients had an excellent/good, one third a fair and another third a poor outcome compared to the preoperative symptoms, while patients’ satisfaction was good in > 70% of the patients. Patients’ satisfaction was not statistically correlated with tumor entity and grading as well as age and gender.

**Table 3:** Postoperative clinical outcome and patients’ satisfaction during follow-up

Characteristics	Timepoint					
	postop (*n* = 40)	3-mo FU (*n* = 40)	12-mo FU (*n* = 40)	24-mo FU (*n* = 36)	last FU (*n* = 40)	‚best ‘ postop status (*n *= 40)
Odom Score						
Excellent, %	10%	21%	7%	4%	10%	21%
Good, %	18%	13%	14%	27%	25%	27%
Fair, %	13%	21%	29%	31%	28%	31%
Poor, %	59%	45%	50%	38%	37%	21%
Patient Satisfaction Index						
I, %	58%	56%	36%	46%	43%	58%
II, %	8%	18%	29%	31%	28%	31%
III, %	5%	5%	0%	4%	5%	10%
IV, %	29%	21%	35%	19%	24%	1%

**Fig. 1:**
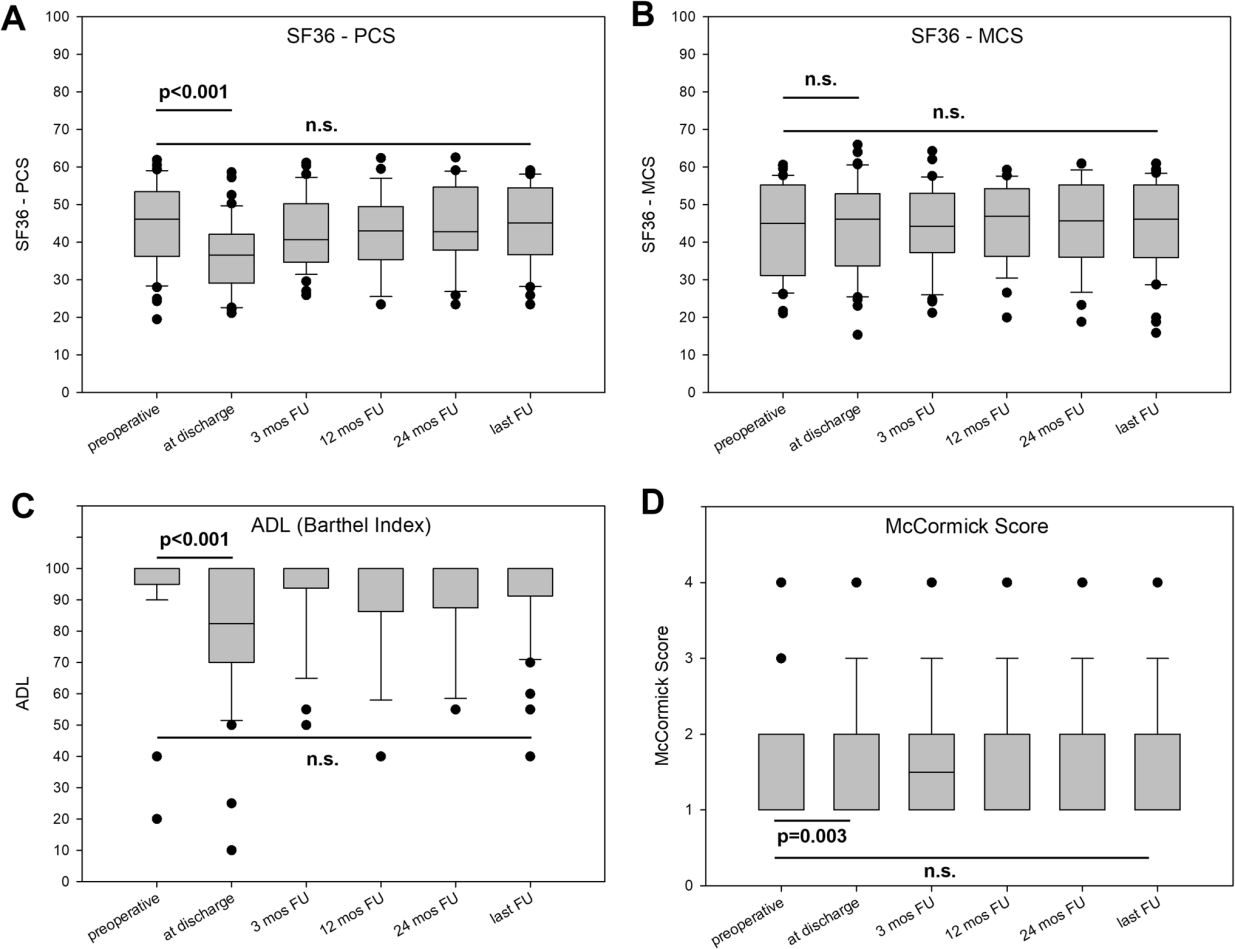
Box plots for (**A**) Physical and (**B**) Mental Compound Score (PCS and MCS) Short Form (SF)−36 Health Survey, **C** Activities of Daily Living (ADL) according to Barthel Index and (**D**) McCormick Score for the preoperative status and during postoperative long-term follow-up. P-values for the preoperative vs. discharge status as well as preoperative vs. last follow-up status are additionally displayed (n.s.: not significant)

QoL and general performance data of the overall cohort preoperatively, at discharge and during follow-up are graphically presented in Fig. 1. Both the physical and mental component of QoL perception (mean SF36-PCS: 45.1 ± 11.1; mean SF36-MCS: 43.3 ± 12.1) were preoperatively slightly reduced compared to the healthy control population (50.0); median BI was 100 (range: 40–100) and median McCormick Score was 1 (range: 1–4). In general, there was a significant decrease in the performance status (BI and McCormick Score) as well as physical component of QoL perception directly after surgery which completely regressed within the first 12 months and stayed stable on the level of the preoperative status/perception during the further follow-up period. The mental component of QoL perception leveled at the intensity of the preoperative perception directly after surgery and during all further postoperative follow-up observations.

At last follow-up, mean SF36-PCS was 44.5 ± 10.4 and mean SF36-MCS was 44.5 ± 12.2; median BI was 100 (range: 40–100) and median McCormick Score was 1 (range: 1–4); there were no significant differences compared to the preoperative status for each item. At the timepoint of the individual patient’s best postoperative status, mean SF36-PCS was 45.3 ± 10.1 and mean SF36-MCS was 45.4 ± 11.6; median BI was 100 (range: 40–100) and median McCormick Score was 1 (range: 1–4)); there were also no significant differences compared to the preoperative status for each item.

The rate of patients with improved, stable and deteriorated QoL perception and general performance status after surgery and during follow-up (compared to the preoperative status) are shown in Table 4. At last follow-up, 30% of the patients had a better score in the physical component of QoL perception and 33% in the mental component of QoL perception compared to the healthy control population. At the timepoint of the individual patient’s best postoperative status, 30% of the patients had a better score in the physical component of QoL perception and 38% in the mental component of QoL perception compared to the healthy control population.

**Table 4:** Detailed postoperative quality-of-life and performance status outcome during follow-up

Change in (compared to the preoperative status)	Timepoint					
	postop (*n* = 40)	3-mo FU (*n* = 40)	12-mo FU (*n *= 40)	24-mo FU (*n* = 36)	last FU (*n* = 40)	‚best ‘ postop status (*n* = 40)
SF-36-PCS						
Improved, %	5%	26%	25%	23%	30%	30%
Stable, %	33%	35%	32%	35%	33%	48%
Deteriorated, %	62%	39%	43%	42%	37%	22%
SF-36-MCS						
Improved, %	28%	26%	32%	35%	33%	38%
Stable, %	35%	42%	36%	35%	35%	48%
Deteriorated, %	37%	32%	32%	30%	32%	14%
ADL (Barthel Index)						
Improved, %	0%	16%	14%	15%	15%	20%
Stable, %	28%	58%	61%	54%	60%	63%
Deteriorated, %	72%	26%	25%	31%	25%	17%
McCormick Score						
Improved, %	8%	21%	25%	31%	28%	30%
Stable, %	23%	55%	49%	46%	44%	53%
Deteriorated, %	69%	24%	26%	23%	28%	17%

Subgroup analysis of detailed neurological, functional and QoL outcome according to the aggressiveness of the tumor entity (stratified in three distinct groups: 1) ependymomas, 2) glial tumors [without ependymomas] and 3) benign non-glial lesions) are displayed in Supplementary Tables 2– 5.

### Prognostic factors for quality-of-life and neurological outcome

Factors potentially associated with a favorable/unfavorable follow-up outcome regarding the physical and mental component of QoL perception in univariate analysis are summarized in Table 5. A permanent postoperatively new or worsened sensory deficit after surgery (OR = 0.15, 95% confidence interval: 0.03–0.81; *p* = 0.027) and a preoperatively higher score in SF36-PCS (OR = 0.92, 95% confidence interval: 0.86–0.99; *p* = 0.018) were significantly associated with a less favorable prognosis for a postoperative improvement in the physical component of QoL perception at last follow-up. This was also true in the multivariate analysis (see Supplementary Table 6). There were no significant associations regarding a favorable last follow-up outcome for the mental component of QoL perception.

**Table 5:** Univariate analysis for factors associated with a favorable prognosis for quality-of life perception at last follow-up after surgery

	SF36-PCS Odds ratio (*p* value/95% CI)	SF36-MCS Odds ratio (*p* value/95% CI)
Univariate		
Age at surgery Per year	0.99 (0.56/0.95–1.03)	0.98 (0.352/0.94–1.02)
Gender Male vs. female	1.17 (0.82/0.32–4.28)	1.14 (0.84/0.31–4.23)
Preoperative SF36-Score Score value	**0.92 (0.02/0.86–0.99)**	0.96 (0.13/0.91–1.01)
Tumor location High cervical vs. low cervical vs. high thoracic vs. low thoracic	0.98 (0.95/0.53–1.82)	0.77 (0.42/0.41–1.44)
Extent of tumor Per level	1.50 (0.41/0.57–3.89)	0.78 (0.60/0.31–1.95)
Tumor entity Histopathological diagnosis	1.02 (0.72/0.91–1.15)	0.98 (0.82/0.88–1.11)
Extent of resection GTR vs. STR vs. biopsy	0.70 (0.52/0.24–2.05)	3.07 (0.17/0.61–15.44)
Permanent postop. new or deteriorated motor deficit Yes vs. no	0.60 (0.53/0.12–2.94)	0.41 (0.27/0.08–2.03)
Permanent postop. new or deteriorated sensory deficit Yes vs. no	**0.15 (0.03/0.03–0.81)**	0.76 (0.71/0.18–3.26)
Permanent postop. new or deteriorated motor deficit Yes vs. no	0.67 (0.56/0.16–2.85)	0.44 (0.28/0.10–1.92)

There were no significant associations between the occurrence of permanent postoperative new or worsened neurological deficits at last follow-up after surgical resection and the following factors: age at surgery (per year), gender (male vs. female), preoperative neurological deficits (yes vs. no), preoperative SF36-Score PCS and MCS (score value), tumor location (high cervical vs. low cervical vs. high thoracic vs. low thoracic), extent of tumor (per level), tumor entity (histopathological diagnosis), extent of resection (GTR vs. STR vs. biopsy) and immediate postoperative neurological deterioration (yes vs. no).

## Discussion

This prospective study was performed to collect data about the improvements and changes in i) the neurological status, ii) daily-life function, and iii) physical & mental QoL perception in the postoperative long-term course after IMSCT resection and to identify factors that might be of influence. We could show that 80% of the patients experienced a postoperative improvement or stabilization in motor function, 25% in sensory function, 75% in gait function and 75% in daily-life activities compared to the preoperative status. QoL perception improved or stabilized in 65% for mental-related aspects and in 63% of the patients for physical-related aspects with presence of a permanent postoperatively new or worsened sensory deficit after surgery and a preoperatively higher score in QoL perception being significant factors of influence for a worse longer-term outcome regarding the physical component of QoL perception.

Resection of IMSCTs is the mainstay of treatment today and enables long-term progression-free survival in the majority of IMSCT entities. While recent advances in neuroimaging, microsurgical techniques, and intraoperative neurophysiological monitoring have greatly facilitated safer and more aggressive resections [15, 17], surgery still poses significant technical challenges and the risk of perioperative and long-term morbidity [6, 8, 16, 18]. So far, several studies have described the outcomes after IMSCT resection, but remain limited due to the retrospective study character and due to the fact that either only gross-functional compound scores (e.g. McCormick scale) were analyzed and/or the follow-up period remained short with 12 months and less [3, 6–8, 16, 22, 27]. In a recent literature review of those studies by Tropeano et al. [22] (including 46 case series) the rate of patients with a McCormick Score of I or II ranged between 40–94% preoperatively and between 44–100% at postoperative follow-up which is comparable with the results of our prospective study (preoperative: 93%; at last follow-up: 88%), underlining the reliability of our data.

However, gross-functional scales might only poorly reflect the global characterization of the patient’s condition and well-being when a deficit occurs [9, 23]. To overcome these limitations, it is essential to specifically address both the detailed neurological as well as fine-motor/-functional outcome, but also and especially include health-related QoL aspects before and after IMSCT resection. So far, larger case series on postoperative QoL aspects after IMSCT resection in adults are scarce and mostly only of retrospective manner lacking preoperative QoL data [20, 23]. However, in the small prospective series of Nakanishi et al. [13] altogether 16 patients were enrolled and the authors could show that QoL (assessed by SF36 Health Survey) remained stable in the overall cohort when comparing the preoperative status with the 6–12 months follow-up status after surgical resection. This is in line with our results underlining the reliability of our data. The authors also found that patients with a preoperative higher grade in functional scoring tended to demonstrate maintenance or deterioration of QoL perception after surgery, while patients with preoperative lower grades in functional scoring tended to demonstrate modest to mild improvement in postoperative QoL aspects at 6–12 months after surgery. In line with this, we could also show that a higher preoperative QoL perception (physical component) was a significant risk factor for a less favourable QoL perception during the postoperative follow-up (completely independent from the neurological follow-up outcome that was not different between patients with a higher and lower preoperative QoL perception). This suggests that patients with a higher level of performance or physical QoL before surgery are less likely to profit of IMSCTs surgery in terms of physical QoL improvement, but have a higher risk for physical QoL deterioration in the postoperative follow-up course (even so this is not true for the mental QoL component). We emphasize to keep in mind that patients with high functioning preoperatively have little room to improve postoperatively but high potential to stabilize or slightly decline due to surgical trauma which might potentially limit this association to a metric rather than a true biological risk factor.

In our overall cohort, QoL aspects (physical and mental components) and ADL did not significantly differ between the preoperative status and the status at longer-term postoperative follow-up on a pooled cohort-wide level. Therefore, contrary to what might have been expected, it seems that pooled QoL does not generally improve after IMSCT resection. However, these results can also be interpreted that resection is indispensable to arrest the preoperatively often rapidly progressive QoL deterioration in patients with IMSCT and enables longer-term stabilization. This would be in line with the results seen in the case series of Xiao et al. [26] where QoL (as measured by the EQ-5D, PDQ, or PHQ-9) also remained stable before and after IMSCT surgery. However, certain patients achieved a QoL improvement and the rate of patients showing an improvement in QoL aspects during postoperative follow-up was identified to be 16–28% in the series of Xiao et al. [26]. This is also in line with our results where the rate of improvement above the threshold of minimal important clinical difference in QoL aspects was 30% (SF36-PCS) and 33% (SF36-MCS) and the rate in ADL improvement was 15%. Altogether, during our postoperative longer-term follow-up, QoL perception improves or stabilizes in 63% of the patients for physical-related aspects, in 65% for mental-related aspects and in 75% for ADL aspects.

Vice versa, this also means that 25–37% show a postoperative decline in QoL and ADL aspects in the follow-up course after IMSCT surgery. It is therefore important to identify those patients who require greater perioperative and long-term attention with regard to QoL optimization. This seems to be especially true for those patients with a permanent postoperatively new or worsened deficit of superficial sensation and fine epicritical sensory function after surgery that was significantly associated with a less favorable prognosis for a postoperative improvement in the physical QoL perception according to our regression analysis, even so this might be dealt with caution due to the limited sample. This is also in line with previous studies that have shown hints that the postoperative course of QoL improvement is linked to the postoperative neurological outcome [20, 23]. This fact underlines the utmost importance of the concept of maximally safe microsurgical tumor resection as well as preservation of dorsal column function as neuroanatomical correlate and pathophysiological mechanism for disturbance of epicritical sensory function. We believe that multimodal IONM can significantly aid the goal of maximal safe surgery, even so causal inferences regarding IONM efficacy cannot be drawn methodologically in this prospective study due to the lack of a non-IONM control group.

Limitations of our study are mainly owed to the single-center design and the limited sample size given the overall very low incidence of intramedullary spinal cord tumors. Moreover, IMSCT entities are not homogeneous and might differ in their natural course. However, tumor entity was not found to be a significant factor in our regression analysis with regard to postoperative longer-term physical QoL outcome, even so this might be dealt with caution as the limited sample size is a risk for overfitting and limited statistical power for regression analysis. However, most important, we want to emphasize that the neurological and functional as well as QoL data collected at the different postoperative follow-up timepoints are always a ‘mixture’ of initial postsurgical deterioration, secondary regain of function in the further postoperative recovery period and a simultaneous deterioration due to a potential tumor-progression or postoperative-/disease-dependent slowly ever-evolving processes (e.g. gliosis formation). Even so it is very difficult to differentiate these concurring aspects from each other, we always additionally report the category of ‘best postoperative status’ for each outcome item as an attempt to address this dilemma and give a clue about what might be more surgery-related or underlying disease-related changes. However, we want to emphasize that the category of ‘best postoperative status’ should always be interpreted with caution as it introduces heterogeneity in outcome timing and may inflate perceived benefits by selectively capturing transient postoperative improvements. It might therefore obscure the true clinical long-term trajectory of individual patients’ outcomes (which might be better defined by fixed-time follow-up intervals as described above), but might help to overcome potential selection and survivorship bias inherent to long-term follow-up studies.

## Conclusion

Our prospective study on detailed follow-up outcome after cervicothoracic IMSCT resection shows that IONM-aided microsurgical resection is safe and enables a high rate of gross-total tumor resection (88%). During longer-term follow-up, 80% of the patients experience a postoperative improved or stable course in motor function, 25% in sensory function and 75% in gait function compared to the preoperative status. QOL perception improves or stabilizes in 63% of the patients for physical-related aspects and in 65% for mental-related aspects. However, presence of a permanent postoperatively new or worsened sensory deficit after surgery and a preoperatively higher score in QoL perception were significant risk factors for a worse longer-term outcome regarding the physical component of QoL perception, but not the mental component of QoL perception.

*OR* Odds ratio, *CI* 95% confidence interval.

## Supplementary Information

Below is the link to the electronic supplementary material.

ESM 1 Supplementary Material 1 (DOCX 29.1 KB)

ESM 2 Supplementary Material 2 (DOCX 35.5 KB)

ESM 3 Supplementary Material 3 (DOCX 36.6 KB)

ESM 4 Supplementary Material 4 (DOCX 33.2 KB)

ESM 5 Supplementary Material 5 (DOCX 35.1 KB)

ESM 6 Supplementary Material 6 (DOCX 27.6 KB)

## Data Availability

The data that support the findings of this study are available from the corresponding author, Sebastian Siller, upon reasonable request.

## Abbreviations

BI: Barthel Index
ca.: Circa
CST: Corticospinal tract
DCM: Dorsal column mapping
D wave: Direct wave
FN: False-negative
FP: False-positive
frEMG: Free-running electromyography
i.a.: Inter alia
IMSCT: Intramedullary spinal cord tumor
IONM: Intraoperative neurophysiological monitoring
KPS: Karnofsky Performance Scale
mJOA: Modified Japanese orthopaedic association score
MRC: Medical Research Council
MRI: Magnetic resonance imaging
mTcMEPs: Muscular transcranially motor evoked potentials
NHPT: Nine-Hole Peg Test
PSS: Performance Status Scale
QoL: Quality of life
SD: Standard deviation
SF36: Short Form (SF)-36v2^®^ Health Survey
SSEPs: Somatosensory evoked potentials
TcMEPs: Transcranially motor evoked potentials
USA: United States of America
WHO: World Health Organization
