# Supplementary material for: Quality-of-life and detailed functional outcome after IONM-aided microsurgical resection of cervical and thoracic intramedullary spinal cord tumors in adults
Source: Acta Neurochir (Wien). 2026 Mar 25;168(1):86. doi: 10.1007/s00701-026-06836-0 (PMC13021858; doi:10.1007/s00701-026-06836-0)
Supplement: Supplementary file 1 — Supplementary Material 1 (DOCX 29.1 KB) [file 701_2026_6836_MOESM1_ESM.docx]

| **SUPPLEMENTARY TABLE 1: Association between significant IONM events during surgery and the presence of postoperatively new or worsened neurological deficits at last follow-up (n = 40)** | | |
| --- | --- | --- |
| New or worsened deficits in  (compared to the preoperative status) | Significant IONM events in | |
|  | TcMEPs | |
| motor function | yes | no |
| yes | 4 | 4 |
| no | 12 | 20 |
|  |  | |
| motor function | D wave | |
|  | yes | no |
| yes | 1 | 7 |
| no | 0 | 32 |
|  |  | |
| motor function | frEMG | |
|  | yes | no |
| yes | 2 | 6 |
| no | 5 | 27 |
|  |  | |
| sensory function | SSEPs | |
|  | yes | no |
| yes | 20 | 9 |
| no | 2 | 9 |
|  |  | |
| gait ataxia (proprioception) | SSEPs | |
|  | yes | no |
| yes | 8 | 2 |
| no | 14 | 16 |
|  | | |
