# Supplementary material for: Quality-of-life and detailed functional outcome after IONM-aided microsurgical resection of cervical and thoracic intramedullary spinal cord tumors in adults
Source: Acta Neurochir (Wien). 2026 Mar 25;168(1):86. doi: 10.1007/s00701-026-06836-0 (PMC13021858; doi:10.1007/s00701-026-06836-0)
Supplement: Supplementary file 2 — Supplementary Material 2 (DOCX 35.5 KB) [file 701_2026_6836_MOESM2_ESM.docx]

| **SUPPLEMENTARY TABLE 2: Detailed postoperative neurological outcome during follow-up in different subgroups** | | | | | | |
| --- | --- | --- | --- | --- | --- | --- |
| Change in  (compared to the preoperative status) | Timepoint | | | | | |
|  | postop. | 3-mo.  FU | 12-mo.  FU | 24-mo.  FU | last  FU | ‚best‘  postop.  status |
| **Ependymoma, WHO grade 2** | **(n = 20)** | **(n = 20)** | **(n = 19)** | **(n = 17)** | **(n = 20)** | **(n = 20)** |
| motor function |  |  |  |  |  |  |
| improved, % | 0% | 10% | 27% | 14% | 10% | 27% |
| stable, % | 60% | 60% | 60% | 65% | 75% | 60% |
| deteriorated, % | 40% | 30% | 13% | 21% | 15% | 13% |
| sensory function |  |  |  |  |  |  |
| improved, % | 5% | 5% | 6% | 14% | 15% | 15% |
| stable, % | 5% | 10% | 13% | 0% | 0% | 13% |
| deteriorated, % | 90% | 85% | 81% | 86% | 85% | 72% |
| gait ataxia (proprioception) |  |  |  |  |  |  |
| improved, % | 10% | 10% | 13% | 7% | 15% | 20% |
| stable, % | 40% | 75% | 62% | 57% | 70% | 65% |
| deteriorated, % | 50% | 15% | 25% | 36% | 15% | 15% |
| bladder/bowl function/continence |  |  |  |  |  |  |
| improved, % | 10% | 20% | 19% | 29% | 20% | 29% |
| stable, % | 90% | 80% | 81% | 71% | 80% | 71% |
| deteriorated, % | 0% | 0% | 0% | 0% | 0% | 0% |
|  |  |  |  |  |  |  |
| **Glioma (except ependymoma), WHO grade 1-4** | **(n = 5)** | **(n = 5)** | **(n = 5)** | **(n = 5)** | **(n = 5)** | **(n = 5)** |
| motor function |  |  |  |  |  |  |
| improved, % | 0% | 40% | 0% | 0% | 0% | 40% |
| stable, % | 80% | 40% | 80% | 100% | 60% | 40% |
| deteriorated, % | 20% | 20% | 20% | 0% | 40% | 20% |
| sensory function |  |  |  |  |  |  |
| improved, % | 0% | 20% | 20% | 0% | 0% | 20% |
| stable, % | 60% | 0% | 40% | 50% | 0% | 40% |
| deteriorated, % | 40% | 80% | 40% | 50% | 100% | 40% |
| gait ataxia (proprioception) |  |  |  |  |  |  |
| improved, % | 20% | 60% | 60% | 50% | 40% | 60% |
| stable, % | 40% | 20% | 0% | 50% | 0% | 20% |
| deteriorated, % | 40% | 20% | 40% | 0% | 60% | 20% |
| bladder/bowl function/continence |  |  |  |  |  |  |
| improved, % | 20% | 20% | 20% | 50% | 20% | 20% |
| stable, % | 80% | 80% | 80% | 50% | 80% | 80% |
| deteriorated, % | 0% | 0% | 0% | 0% | 0% | 0% |
|  |  |  |  |  |  |  |
| **Non-Glial Benign Lesions, WHO grade 1** | **(n = 15)** | **(n = 15)** | **(n = 15)** | **(n = 14)** | **(n = 15)** | **(n = 15)** |
| motor function |  |  |  |  |  |  |
| improved, % | 13% | 23% | 50% | 10% | 27% | 27% |
| stable, % | 54% | 54% | 38% | 50% | 53% | 61% |
| deteriorated, % | 33% | 23% | 12% | 40% | 20% | 12% |
| sensory function |  |  |  |  |  |  |
| improved, % | 13% | 46% | 18% | 20% | 33% | 46% |
| stable, % | 3% | 0% | 21% | 10% | 13% | 13% |
| deteriorated, % | 54% | 54% | 61% | 70% | 54% | 41% |
| gait ataxia (proprioception) |  |  |  |  |  |  |
| improved, % | 7% | 23% | 25% | 30% | 20% | 27% |
| stable, % | 60% | 54% | 50% | 40% | 53% | 60% |
| deteriorated, % | 33% | 23% | 25% | 30% | 27% | 13% |
| bladder/bowl function/continence |  |  |  |  |  |  |
| improved, % | 13% | 23% | 13% | 10% | 7% | 20% |
| stable, % | 80% | 77% | 87% | 90% | 93% | 80% |
| deteriorated, % | 7% | 0% | 0% | 0% | 0% | 0% |
|  | | | | | | |
