# Supplementary material for: Quality-of-life and detailed functional outcome after IONM-aided microsurgical resection of cervical and thoracic intramedullary spinal cord tumors in adults
Source: Acta Neurochir (Wien). 2026 Mar 25;168(1):86. doi: 10.1007/s00701-026-06836-0 (PMC13021858; doi:10.1007/s00701-026-06836-0)
Supplement: Supplementary file 3 — Supplementary Material 3 (DOCX 36.6 KB) [file 701_2026_6836_MOESM3_ESM.docx]

| **SUPPLEMENTARY TABLE 3: Postoperative clinical outcome and patients’ satisfaction during follow-up in different subgroups** | | | | | | |
| --- | --- | --- | --- | --- | --- | --- |
| Characteristics | Timepoint | | | | | |
|  | postop. | 3-mo.  FU | 12-mo.  FU | 24-mo.  FU | last  FU | ‚best‘  postop.  status |
| **Ependymoma, WHO grade 2** | **(n = 20)** | **(n = 20)** | **(n = 19)** | **(n = 17)** | **(n = 20)** | **(n = 20)** |
| Odom Score |  |  |  |  |  |  |
| excellent, % | 5% | 25% | 7% | 0% | 5% | 25% |
| good, % | 10% | 15% | 14% | 21% | 20% | 20% |
| fair, % | 15% | 15% | 36% | 43% | 40% | 40% |
| poor, % | 70% | 45% | 43% | 36% | 35% | 15% |
| Patient Satisfaction Index |  |  |  |  |  |  |
| I, % | 60% | 50% | 43% | 50% | 40% | 40% |
| II, % | 0% | 30% | 36% | 21% | 25% | 35% |
| III, % | 0% | 5% | 0% | 8% | 10% | 10% |
| IV, % | 40% | 15% | 21% | 21% | 25% | 15% |
|  |  |  |  |  |  |  |
| **Glioma (except ependymoma), WHO grade 1-4** | **(n = 5)** | **(n = 5)** | **(n = 5)** | **(n = 4)** | **(n = 5)** | **(n = 5)** |
| Odom Score |  |  |  |  |  |  |
| excellent, % | 0% | 0% | 0% | 0% | 0% | 0% |
| good, % | 0% | 0% | 0% | 0% | 20% | 20% |
| fair, % | 0% | 20% | 40% | 50% | 20% | 40% |
| poor, % | 100% | 80% | 60% | 50% | 60% | 40% |
| Patient Satisfaction Index |  |  |  |  |  |  |
| I, % | 40% | 40% | 20% | 50% | 20% | 40% |
| II, % | 20% | 0% | 0% | 0% | 20% | 20% |
| III, % | 0% | 20% | 0% | 0% | 0% | 0% |
| IV, % | 40% | 40% | 80% | 50% | 60% | 40% |
|  |  |  |  |  |  |  |
| **Non-Glial Benign Lesions, WHO grade 1** | **(n = 15)** | **(n = 15)** | **(n = 15)** | **(n = 15)** | **(n = 15)** | **(n = 15)** |
| Odom Score |  |  |  |  |  |  |
| excellent, % | 20% | 23% | 13% | 10% | 20% | 23% |
| good, % | 27% | 15% | 25% | 40% | 33% | 15% |
| fair, % | 13% | 31% | 12% | 10% | 13% | 31% |
| poor, % | 60% | 31% | 50% | 40% | 34% | 31% |
| Patient Satisfaction Index |  |  |  |  |  |  |
| I, % | 67% | 69% | 38% | 40% | 53% | 53% |
| II, % | 13% | 8% | 38% | 50% | 34% | 7% |
| III, % | 13% | 0% | 0% | 0% | 0% | 13% |
| IV, % | 7% | 23% | 24% | 10% | 13% | 7% |
|  | | | | | | |
