# Supplementary material for: Quality-of-life and detailed functional outcome after IONM-aided microsurgical resection of cervical and thoracic intramedullary spinal cord tumors in adults
Source: Acta Neurochir (Wien). 2026 Mar 25;168(1):86. doi: 10.1007/s00701-026-06836-0 (PMC13021858; doi:10.1007/s00701-026-06836-0)
Supplement: Supplementary file 4 — Supplementary Material 4 (DOCX 33.2 KB) [file 701_2026_6836_MOESM4_ESM.docx]

| **SUPPLEMENTARY TABLE 4: Overall preoperative and postoperative quality-of-life and performance status outcome during follow-up in different subgroups*** | | | | | | | |
| --- | --- | --- | --- | --- | --- | --- | --- |
| Change in  (compared to the preoperative status) | Timepoint | | | | | | |
|  | preop. | postop. | 3-mo.  FU | 12-mo.  FU | 24-mo.  FU | last  FU | ‚best‘  postop.  status |
| **Ependymoma, WHO grade 2** | **(n = 20)** | **(n = 20)** | **(n = 20)** | **(n = 19)** | **(n = 17)** | **(n = 20)** | **(n = 20)** |
| mean SF-36-PCS | 45.8  ± 9.3 | 35.5  ± 8.5 | 39.9  ± 7.6 | 41.3  ± 7.8 | 43.8  ± 10.1 | 43.2  ± 8.3 | 43.9  ± 8.0 |
| mean SF-36-MCS | 44.4  ± 12.4 | 44.3  ± 11.9 | 44.7  ± 11.4 | 45.7  ± 10.0 | 45.1  ± 12.9 | 44.6  ± 13.5 | 47.2  ± 12.2 |
| median ADL (Barthel Index) [range] | 100  [20-100] | 75  [10-100] | 100  [50-100] | 100  [40-100] | 100  [55-100] | 100  [55-100] | 100  [40-100] |
| median McCormick Score [range] | 1  [1-3] | 2  [1-4] | 1  [1-4] | 1  [1-3] | 2  [1-4] | 1  [1-4] | 1  [1-4] |
|  |  |  |  |  |  |  |  |
| **Glioma (except ependymoma), WHO grade 1-4** | **(n = 5)** | **(n = 5)** | **(n = 5)** | **(n = 5)** | **(n =4)** | **(n = 5)** | **(n = 5)** |
| mean SF-36-PCS | 39.9  ± 11.3 | 29.1  ± 7.6 | 40.2  ± 9.6 | 37.3  ± 9.7 | 35.2  ± 11.1 | 38.0  ± 10.2 | 40.7  ± 10.1 |
| mean SF-36-MCS | 38.8  ± 11.7 | 34.0  ± 11.7 | 38.6  ± 11.2 | 42.9  ± 8.9 | 34.1  ± 15.3 | 40.1  ± 9.5 | 38.4  ± 11.1 |
| median ADL (Barthel Index) [range] | 100  [40-100] | 75  [25-100] | 100  [65-100] | 100  [40-100] | 80  [55-100] | 100  [40-100] | 100  [65-100] |
| median McCormick Score [range] | 2  [1-4] | 2  [2-4] | 2  [1-4] | 2  [1-4] | 2  [1-2] | 2  [1-4] | 2  [1-4] |
|  |  |  |  |  |  |  |  |
| **Non-Glial Benign Lesions, WHO grade 1** | **(n = 15)** | **(n = 15)** | **(n = 15)** | **(n = 15)** | **(n = 15)** | **(n = 15)** | **(n = 15)** |
| mean SF-36-PCS | 45.8  ± 9.3 | 38.8  ± 9.9 | 47.5  ± 11.2 | 47.3  ± 13.4 | 46.6  ± 13.2 | 48.4  ± 12.1 | 49.7  ± 10.6 |
| mean SF-36-MCS | 43.3  ± 11.7 | 46.8  ± 13.0 | 45.6  ± 10.5 | 44.0  ± 13.0 | 45.7  ± 10.2 | 45.8  ± 11.5 | 45.3  ± 10.7 |
| median ADL (Barthel Index) [range] | 100  [90-100] | 95  [65-100] | 100  [55-100] | 100  [60-100] | 80  [75-100] | 100  [70-100] | 100  [90-100] |
| median McCormick Score [range] | 1  [1-2] | 2  [1-2] | 1  [1-2] | 2  [1-3] | 2  [1-3] | 1  [1-3] | 1  [1-2] |
| * Mean values are presented ± standard deviation. | | | | | | | |
