# Supplementary material for: Quality-of-life and detailed functional outcome after IONM-aided microsurgical resection of cervical and thoracic intramedullary spinal cord tumors in adults
Source: Acta Neurochir (Wien). 2026 Mar 25;168(1):86. doi: 10.1007/s00701-026-06836-0 (PMC13021858; doi:10.1007/s00701-026-06836-0)
Supplement: Supplementary file 6 — Supplementary Material 6 (DOCX 27.6 KB) [file 701_2026_6836_MOESM6_ESM.docx]

| **SUPPLEMENTARY TABLE 6: Multivariate analysis for factors associated with a favorable prognosis for quality-of life perception at last follow-up after surgery** | | |
| --- | --- | --- |
|  | **SF36-PCS**  Odds ratio  (p value / 95% CI) | **SF36-MCS**  Odds ratio  (p value / 95% CI) |
| **Multivariate** |  |  |
| **preoperative SF36-Score**  score value | **0.90 (0.01 / 0.83-0.98)** | 0.95 (0.09 / 0.90-1.01) |
| **permanent postop. new or deteriorated sensory deficit**  yes vs. no | **0.08 (0.01 / 0.01-0.59)** | 0.48 (0.36 / 0.10-2.37) |
| OR: odds ratio, CI: 95% confidence interval. | | |
